# De Novo Skin Neoplasms in Liver-Transplanted Patients: Single-Center Prospective Evaluation of 105 Cases

**DOI:** 10.3390/medicina58101444

**Published:** 2022-10-13

**Authors:** Alessia Paganelli, Paolo Magistri, Shaniko Kaleci, Johanna Chester, Claudia Pezzini, Barbara Catellani, Silvana Ciardo, Alice Casari, Francesca Giusti, Sara Bassoli, Stefano Di Sandro, Giovanni Pellacani, Francesca Farnetani, Fabrizio Di Benedetto

**Affiliations:** 1 Clinical and Experimental Medicine Ph.D. Program, University of Modena and Reggio Emilia, 41124 Modena, Italy; 2 Dermatology Unit, Modena University Hospital, University of Modena and Reggio Emilia, 41124 Modena, Italy; 3 Hepato-Pancreato-Biliary Surgery and Liver Transplantation Unit, Modena University Hospital, University of Modena and Reggio Emilia, 41124 Modena, Italy; 4 Dermatology Clinic, Department of Clinical Internal, Anesthesiological and Cardiovascular Sciences, Sapienza University of Rome, 00161 Rome, Italy

**Keywords:** liver transplant, LTR, immunosuppression, skin cancer, NMSC, risk factor, AK, SOTR, melanoma, cutaneous oncology

## Abstract

*Background and Objectives*: Solid-organ transplant recipients (SOTRs) are notably considered at risk for developing cutaneous malignancies. However, most of the existing literature is focused on kidney transplant-related non-melanoma skin cancers (NMSCs). Conflicting data have been published so far on NMSC incidence among liver transplant recipients (LTRs), and whether LTRs really should be considered at lower risk remains controversial. The aim of the present study was to prospectively collect data on the incidence of cutaneous neoplasms in an LTR cohort. *Materials and Methods:* All LTRs transplanted at the Hepato-Pancreato-Biliary Surgery and Liver Transplantation Unit of Modena University Hospital from October 2015 to June 2021 underwent a post-transplant periodic skin check at the Dermatology Unit according to our institutional integrated care pathway. Data on the presence of cutaneous malignant and premalignant lesions were collected at every timepoint. *Results:* A total of 105 patients were enrolled in the present study. Nearly 15% of the patients developed cutaneous cancerous and/or precancerous lesions during the follow-up period. Almost half of the skin cancerous lesions were basal cell carcinomas. Actinic keratoses (AKs) were observed in six patients. Four patients developed in situ squamous cell carcinomas, and one patient was diagnosed with stage I malignant melanoma. Otherwise, well-established risk factors for the occurrence of skin tumors, such as skin phototype, cumulative sun exposure, and familial history of cutaneous neoplasms, seemed to have no direct impact on skin cancer occurrence in our cohort, as well as an immunosuppressive regimen and the occurrence of non-cutaneous neoplasms. *Conclusions*: Close dermatological follow-up is crucial for LTRs, and shared protocols of regular skin checks in this particular subset of patients are needed in transplant centers.

## 1. Introduction

Solid-organ transplant recipients (SOTRs) are known to be particularly susceptible to the development of malignancies, with skin cancers accounting for 40% of the total SOT-associated neoplasms [1,2,3,4]. In particular, non-melanoma skin cancers (NMSCs) seem to occur very frequently in SOTRs, representing one of the most common post-transplant complications. Current research proposes increased risk estimates as high as 80-fold for squamous cell carcinoma (SCC) and 16-fold for basal cell carcinoma (BCC) compared to the general population [1].

However, some authors reported lower rates of skin cancer among liver transplant recipients (LTRs) compared to other SOTRs, and whether LTRs really should be considered at risk remains controversial [5]. Most of the published data are retrospective in nature and do not always take into account the patient baseline characteristics at the time of transplantation, therefore leading to the high variability of the available information. 

Despite the recommendation among most protocols for a complete dermatological assessment for all future transplant recipients, most of the available data are retrospective and do not always take into account the patient baseline characteristics at the time of transplantation. 

Therefore, whether LTRs really should be considered at lower risk for skin tumors remains controversial. We report the incidence of de novo skin neoplastic and pre-neoplastic lesions among a consecutive cohort of all LTRs treated between October 2015 and June 2021 at our center. The risk factors associated with the development of skin neoplasms are also reported.

## 2. Materials and Methods

We prospectively collected data on the incidence of de novo skin neoplastic and pre-neoplastic lesions among a cohort of LTRs transplanted at the Hepato-Pancreato-Biliary Surgery and Liver Transplantation Unit and followed-up at the Dermatology Unit between October 2015 and December 2021 at Modena University Hospital. Each patient was prescribed a baseline dermatological visit at the time of liver transplantation (T0) and subsequent follow-up after 3 months (T1), every six months for 2 years (T2-T4), and annually thereafter, unless altered by the visiting dermatologist. The study protocol was conducted according to our institutional integrated care pathway and in accordance with the principles of the Declaration of Helsinki. Statistical analysis was performed with STATA software version 17 (StataCorp. 2021). The chi-square test (Fisher’s exact test) was used to examine the relationships between qualitative variables. Student’s t-test was used to assess differences for continuous variables between groups. The incidence of de novo lesions was performed with Kaplan–Meier curves, with a log-rank test for comparison between the curves. A *p*-value of <0.05 was considered significant.

We collected complete baseline data at our initial clinical examination, including demographic information, comorbidities, post-transplant course, immunosuppressant medications, and dermatological characteristics, which included the familial and/or personal history of previous skin neoplasms and the presence/absence of any existing skin lesions. Despite not being an established cause of liver failure, diabetes was considered a baseline significant comorbidity due to both diabetes-related immunosuppression and its possible association with metabolic syndrome and/or non-alcoholic fatty liver disease. 

Dermatological follow-up included total body clinical and dermoscopic examination for the detection of any de novo cutaneous lesions. Reflectance confocal microscopy may have been employed in case of diagnostic uncertainty.

The diagnosis of actinic keratosis (AK) and superficial BCC was often based on non-invasive imaging criteria only (no histopathological confirmation), whereas for lesions in which non-superficial BCC, SCC, or melanoma was suspected, full surgical excision with histopathological confirmation was performed. 

## 3. Results

A total of 105 patients were enrolled in our study. Nearly two-thirds of the LTRs transplanted at our center did not undergo dermatological evaluation and were therefore excluded from the present study (215/320). Most patients were not completely compliant with the exact protocol time schedule, mainly due to the COVID-19 pandemic. The patient demographic information, comorbidities, post-transplant course, immunosuppressant regimen, and dermatological characteristics are described in Table 1.

**Figure 1:**
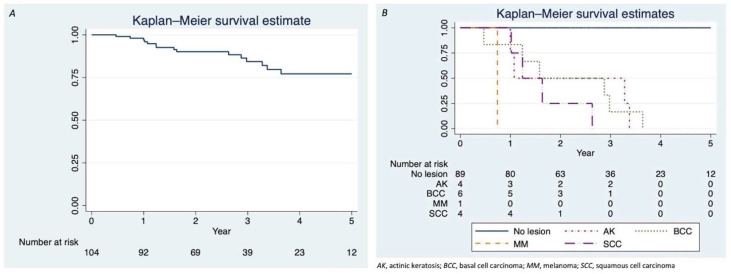
Disease-free survival in the LTR cohort. (**A**) Five-year disease-free overall survival represented through Kaplan–Meier curve. Actinic keratosis, melanoma, and basal and squamous cell carcinoma were all considered events. (**B**) Five-year disease-free survival for different subgroups of patients based on the type of occurring lesion represented through Kaplan–Meier curve.

The mean follow-up was 2.8 years (±1.6, range 0.4–6.2). Nearly 14% (*n* = 15; 14.3%) of patients developed cutaneous malignant and/or pre-malignant lesions during the follow-up period (see Table 2), with more intensive dermatological follow-up associated with such findings (a mean of 5.5 vs. 3.4 dermatological visits; *p*=0.003). Most of the lesions occurred during the first three years after liver transplant (see Figure 1), without any new cutaneous neoplasms detected at the longer term (4/5 years follow-up).

Malignant melanoma was detected in one patient only. Nearly half of the patients affected by malignant and/or pre-malignant lesions developed BCCs (*n* = 6), two of which were classified as superficial. In total, four patients were diagnosed with in situ SCC (or Bowen disease). The mean age at NMSC diagnosis was 58 years. Importantly, no cutaneous malignancies with locoregional or distant spreading were observed during the study follow-up period. AKs were detected in six patients, two of whom also developed SCCs.

Observations of non-malignant cutaneous lesions included viral warts and/or condylomas (*n* = 10), cutaneous drug-induced reactions (*n* = 4), bacterial folliculitis (*n* = 3), recurrent pityriasis versicolor (*n* = 2), and fungal intertrigo (*n* = 2). 

## 4. Discussion

Our data do not show any significant association between the occurrence of cutaneous neoplasms and the presence of specific otherwise well-established risk factors among the LTR population (e.g., sun exposure, familial history, age, skin phototype). However, the mean age at NMSC diagnosis largely differed from the general population (58 vs. 71), despite age not being a statistically relevant risk factor among the LTR cohort [6]. In addition, the role of skin phototype in the NMSC risk for SOTRs remains unclear, as most published studies have included white patients only [7,8,9]. A recent study on 96 dark-skinned LTRs reported no NMSC to be detected during a 2-year follow-up, therefore suggesting a significantly lower risk for darker phototypes [10]. Moreover, in a large prospective study from Iannacone et al., typical NMSC risk factors (ethnicity, skin phototype, previous history of NMSC) were associated with NMSC occurrence after transplantation when considering both liver and kidney transplant recipients [11]. Interestingly, the authors found such risk factors were more frequent in subjects requiring renal transplant [11]. Probably further enlargement of our casuistry is needed to better clarify the impact of UV damage, phototype, and genetic predisposition on the occurrence of skin cancers in LTRs. 

Moreover, comorbidities, post-surgical complications, and even immunosuppressive regimens did not seem to have a direct impact on the development of skin malignancies. 

Previous authors have hypothesized that the relatively lower rates of NMSCs in LTRs may depend on the type of immunosuppressants used in such subsets of patients, possibly being associated with lower doses of immune suppression [5,8,12,13,14]. However, no significant links between induction therapy, corticosteroid use, an immunosuppressive regimen, and the development of malignant and/or pre-malignant cutaneous lesions emerge from our data.

Some authors have reported relatively high frequencies of multiple and/or subsequent NMSCs in LTRs [15,16]. On the contrary, we did not detect cases of multiple, de novo NMSCs in almost 3 years of follow-up. 

Rates of around 19% for the occurrence of NMSCs were reported in a French study that included 11,000 SOTRs followed-up over a 3.5-year period, without any systematic dermatological screening [17]. Conversely, we report that less than 10% of the patients developed NMSCs. Our results are in line with other studies demonstrating systematic skin check-ups to be associated with the detection of more preneoplastic lesions [18,19]. 

Finally, our study emphasizes the importance of dermatological follow-up for the correct diagnosis and treatment of non-oncological dermatological comorbidities among LTRs, especially infectious and drug-related dermatoses, which are notably likely to occur in this subset of patients [20,21].

## 5. Conclusions

Our data indicate regular dermatological visits to be more likely associated with the detection of pre-cancerous lesions and/or very localized forms of cutaneous neoplasms. Close dermatologic follow-up after liver transplantation seemed to increase the chances of early NMSC diagnosis and should be integrated into management protocols, even with new-generation immunosuppressant therapies. The present study also suggests that LTRs could be predisposed to develop skin cancer independently of common risk factors, such as skin phototype, age, immunosuppressive regimen, and other non-skin cancer incidence. Future studies aimed at exploring possible immunological mechanisms underlying skin cancer occurrence in SOTRs are warmly encouraged in order to clarify whether NMSC risk in LTRs could at least be partially explained by factors other than immunosuppressants, either transplant-related or drug-related. 

## Figures and Tables

**Table 1:** Patient demographics and clinical data. Data are expressed as absolute numbers and percentages (*n* (%)), unless otherwise specified.

Variable	Total	Patients with Malignant or Pre-Malignant Skin Lesions		
	*n* = 105	No (90, 85.7%)	Yes (15, 14.3%)	*p*-Value
Mean age, yrs. ± SD (range)	55.7 ± 9.5 (26–75)	55.1 ± 9.6 (25.9–75.1)	59.3 ± 8.2 (40.2–68.1)	0.110
Sex, male	82 (78.1)	69 (76.7)	13 (86.7)	0.386
**Comorbidities at baseline:**				
Autoimmune hepatitis	11 (10.5)	10 (11.1)	1 (6.7)	0.697
HCV	30 (28.6)	26 (28.9)	4 (26.7)	0.915
HBV	16 (15.2)	12 (13.3)	4 (26.7)	0.102
Alcoholic hepatitis	17 (16.2)	16 (17.8)	1 (6.7)	0.333
Diabetes	14 (13.3)	11 (12.2)	3 (20.0)	0.302
MELD at transplantation, mean ± SD (range)	16.6 ± 8.2(0–35)	16.6 ± 8.2 (0–35)	16.1 ± 8.4 (0–28)	0.827
Charlson Comorbidity Index	4.3 ± 1.0(2–7)	4.3 ± 1.1 (2–7)	4.0 ±0.8 (3–5)	0.417
Previous malignancies	7 (6.7)	6 (6.7)	1 (6.7)	0.872
**Post-transplant course:**				
ICU stay, mean days ± SD (range)	2.4 ± 4.3 (1–40)	2.6 ± 4.6 (1–40)	1.3 ± 0.6 (1–3)	0.447
Hospital stay, mean days ± SD (range)	19.2 ± 19.3 (5–157)	19.8 ± 20.4 (5–157)	14.8 ± 9.3 (5–37)	0.354
Re-hospitalization within the first month	11 (10.5)	8 (8.9)	3 (20.0)	0.135
Comprehensive Complication Index, mean ± SD (range)	41.3 ± 24.2 (0–100)	41.8 ± 25.2 (0–100)	40.3 ± 16.9 (0–65.5)	0.880
Non-cutaneous post-transplant malignancies	8 (7.6)	7 (7.8)	1 (6.7)	0.979
**Immunosuppressive Therapies:**				
BASILIXIMAB induction therapy	7 (6.7)	7 (7.8)	0 (0.0)	0.290
Corticosteroids *	82 (97.6)	73 (81.1)	9 (60.0)	0.092
Steroideal therapy duration, mean months ± SD (range)	2.1 ± 1.4 (0–6)	2.2 ± 1.5 (0–6)	2.0 ± 1.2 (0–5)	0.683
Tacrolimus *	73 (87.9)	64 (71.1)	9 (60.0)	0.832
Tacrolimus level * medium ± SD (range)	6.9 ± 1.8 (1.5–10.6)	6.9 ± 1.8 (1.5–10.6)	7.1 ± 2.1 (2.9–10.3)	0.773
Everolimus *	13 (12.4)	11 (12.2)	2 (13.3)	0.673
Everolimus level * medium ± SD (range)	0.9 ± 2.4 (0–9.9)	0.9 ± 2.4 (0.9.9)	1.0 ± 2.2 (0.0–6.4)	0.963
Advagraf *	14 (13.3)	13 (14.4)	1 (6.7)	0.547
Mycophenolate mofetil *	7 (6.7)	6 (6.7)	1 (6.7)	0.839
Rapamycin *	1 (0.29)	1 (1.1)	0 (0.0)	0.712
**Baseline Dermatological characteristics**				
Skin Phototype				
II	25 (23.8)	22 (24.4)	3 (20.0)	0.925
III	67 (63.8)	57 (63.3)	10 (66.7)	
IV	10 (9.5)	8 (8.9)	2 (13.3)	
V	1 (0.9)	1 (1.1)	0 (0.0)	
VI	2 (1.9)	2 (2.2)	0 (0.0)	
Days of annual summer sun exposure, mean ± SD (range)	21.9 ± 8.7 (0–45)	21.5 ± 9.0 (0–45)	24.3 ± 7.2 (15–30)	0.253
Familial history of skin neoplasms	3 (2.9)	3 (3.3)	0 (0.0)	0.422
Previous skin neoplasms	0 (0.0)	0 (0.0)	0 (0.0)	-
Non-melanocytic lesion	4.6 ± 11.7 (0–50)	4.2 ± 11.4 (0–50)	7.4 ± 13.7 (0–40)	0.326
Nevi, mean ± SD (range)	20.6 ± 14.0(0–50)	20.5 ± 14.3 (0–50)	21.0 ± 12.9 (5–50)	0.911
Atypical nevi				
0	101 (96.2)	86 (95.6)	15 (100)	0.707
1	3 (2.9)	3 (3.3)	0 (0.0)	
2	1 (0.9)	1 (1.1)	0 (0.0)	

*n*, number; HCV, hepatitis C virus; HBV, hepatitis B virus; MELD, model for end-stage liver disease; ICU, intensive care unit; * in the first month following transplantation.

**Table 2:** Duration of follow-up and patient characteristics. Only patients who developed cancerous or precancerous skin lesions are included in this table.

	Malignant Skin Lesions			Pre-Malignant Skin Lesions	
Patient Data	Melanoma	Squamous Cell Carcinoma	Basal Cell Carcinoma	Actinic Keratosis	*p*-Value
N, % of cohort	1 (0.9)	4 (3.8)	6 (5.7)	6 (5.7)	
Age, *n*, mean yrs. *±* SD (range)	68.1	51.8 ± 9.5 (40.2–63.0)	62.7 ± 5.2 (56.5–67.9)	58.5 ± 7.2 (49.4–67.0)	0.116
Sex, *M*	1 (100)	3 (75.0)	5 (83.3)	5 (83.3)	0.946
Skin phototype					
II	0 (0.0)	0 (0.0)	3 (50.0)	0 (0.0)	0.169
III	1 (100)	3 (75.0)	2 (33.3)	6 (100)	
IV	0 (0.0)	1 (25.0)	1 (16.7)	0 (0.0)	
Time from transplant to lesion observation, months ± SD (range)	8.8	19.5 ± 8.6 (12–31)	25.5 ± 14.6 (5.6–43.7)	25.2 ± 13.5.1 (11.9–40.5)	0.613

## Data Availability

Data are available from the authors upon reasonable request.
